# Advances in an *In Vitro* Tuberculosis Infection Model Using Human Lung Organoids for Host-Directed Therapies

**DOI:** 10.1371/journal.ppat.1012295

**Published:** 2024-07-25

**Authors:** Seung-Yeon Kim, Ji-Ae Choi, Seri Choi, Kee K. Kim, Chang-Hwa Song, Eun-Mi Kim

**Affiliations:** 1 Department of Predictive Toxicology, Korea Institute of Toxicology, Daejeon, Republic of Korea; 2 Department of Biochemistry, College of Natural Sciences, Chungnam National University Daejeon, Republic of Korea; 3 Department of Microbiology, College of Medicine, Chungnam National University, Daejeon, Republic of Korea; 4 Department of Medical Science, College of Medicine, Chungnam National University, Daejeon, Republic of Korea; 5 Translational Immunology Institute, Chungnam National University, Daejeon, Republic of Korea; 6 Korea Bioactive Natural Material Bank, Research Institute of Pharmaceutical Sciences College of Pharmacy, Seoul National University, Seoul, Republic of Korea; 7 Department of Bio & Environmental Technology, College of Science and Convergence Technology, Seoul Women’s University, Seoul, Republic of Korea; Portland VA Medical Center, Oregon Health and Science University, UNITED STATES OF AMERICA

## Abstract

The emergence of drug-resistant *Mycobacterium tuberculosis* (*M*.*tb*) has led to the development of novel anti-tuberculosis (anti-TB) drugs. Common methods for testing the efficacy of new drugs, including two-dimensional cell culture models or animal models, have several limitations. Therefore, an appropriate model representative of the human organism is required. Here, we developed an *M*.*tb* infection model using human lung organoids (hLOs) and demonstrated that *M*.*tb* H37Rv can infect lung epithelial cells and human macrophages (hMφs) in hLOs. This novel *M*.*tb* infection model can be cultured long-term and split several times while maintaining a similar number of *M*.*tb* H37Rv inside the hLOs. Anti-TB drugs reduced the intracellular survival of *M*.*tb* in hLOs. Notably, *M*.*tb* growth in hLOs was effectively suppressed at each passage by rifampicin and bedaquiline. Furthermore, a reduction in inflammatory cytokine production and intracellular survival of *M*.*tb* were observed upon knockdown of MFN2 and HERPUD1 (host-directed therapeutic targets for TB) in our *M*.*tb* H37Rv-infected hLO model. Thus, the incorporation of hMφs and *M*.*tb* into hLOs provides a powerful strategy for generating an *M*.*tb* infection model. This model can effectively reflect host-pathogen interactions and be utilized to test the efficacy of anti-TB drugs and host-directed therapies.

## Author summary

Establishment of *M*.*tb* infection model is imperative to develop new anti-TB drugs based on the pathogenesis of TB. Various animal models, including mice, rats, guinea pigs, non-human primates, rabbits, cattle, and zebrafish, are commonly used in TB research to mimic TB symptoms and study immune responses to *M*.*tb* infection. *In vitro* models, such as agent-based models allow examination of host-pathogen interactions, early granuloma formation and drug screening, providing cellular-level insights. However, these models may not fully represent human immunopathology owing to differences in immune cell distributions. Lung organoids mimic human lung dynamics and functions, providing crucial insights into immune responses to TB. In this study, an *M*.*tb* infection model developed using hLOs demonstrated infection of lung epithelial cells and human macrophages, reflecting host-pathogen interactions. This model is attractive for evaluating the efficacy of anti-TB drugs and host-directed therapies.

## Introduction

Tuberculosis (TB), caused by *Mycobacterium tuberculosis* (*M*.*tb*), is one of the most prevalent bacterial infectious diseases worldwide [1]. Drug resistance presents an urgent and challenging obstacle in the treatment of TB. To address these issues, it is crucial to establish an appropriate model for the development of novel anti-TB drugs.

The primary sites of *M*.*tb* infection are the lungs, with transmission commonly occurring through the alveolar sacs following inhalation. *M*.*tb* primarily targets macrophages, particularly resident alveolar macrophages (AMs), which leading to the formation of granulomas comprising various immune cells [2]. During infection, the alveolar epithelium serves as a harbor for bacterial recognition and uptake, directly interacting with AMs to regulate cytokine expression in response to pathogens [3]. Alveolar epithelial cells (AECs) also provide a niche for *M*.*tb* replication and dissemination during infection [4].

The treatment and diagnosis of susceptible TB have remained unchanged over the last 40 years. In 2019, a new regimen, combination therapy with bedaquiline and linezolid, was approved to treat adults with drug-resistant TB [5]; however new drugs and treatments are still warranted as the emergence of drug-resistant TB continues to increase. *In vitro* cell culture models using mouse macrophages and *in vivo* animal models are commonly used to investigate the pathology and pathogenesis of TB and screen new drugs [6–8]. Despite the well-known similarities between mice and humans, these systems have several limitations, such as variations in the lung lobe structure and lung cell composition [9]. Furthermore, as animals are not natural reservoirs for *M*.*tb*, they only partially mimic the clinical symptoms and immunological indicators of TB, exhibiting challenges in granuloma formation and differing susceptibilities to TB compared to those observed in humans [10]. Therefore, the evaluation results using animal models for the development and application of new TB treatment drugs are less reliable. To address these limitations, it is imperative to develop a novel model utilizing human-derived cells for anti-TB drug testing.

Recently, infection models have been developed to mimic the complexity of the lung microenvironment, offering an alternative to immortal cell lines of lung epithelia and animal models [10,11]. Several models have been developed using advanced cell culture systems to prevent or treat infections. For viral [12] and *M*.*tb* [13] infections, a lung-on-chip model was developed that could regulate the dynamic flow and mimic breathing-like movements. Additionally, host immune responses to Mycobacteria infection were studied using human bronchiolar airway organoids [14]. Furthermore, an *M*.*tb* infection model comprising primary human blood mononuclear cells was developed using a microfluidic plate [15]. In another study, *in vitro* formation of granuloma-like cell aggregates was achieved by utilizing peripheral blood mononuclear cells isolated from patients with latent TB infection and beads coated with purified protein derivatives to mimic *in vivo* behavior of granuloma [16].

Although advanced cell culture systems offer advantages in modeling infectious diseases, they fail to accurately replicate the three-dimensional (3D) structure of the lungs. Human lung organoids (hLOs), which replicate the cellular composition and function of the lungs, have emerged as outstanding models for investigating pathophysiology and drug screening. For establishing severe acute respiratory syndrome coronavirus 2 infection models, human distal lung organoids consisting of AEC2 and basal cells were generated [17]. However, lung models employing human-derived cells for anti-TB drug testing have not been fully developed.

In this study, we generated 3D hLOs derived from human pluripotent stem cells (hPSCs) that exhibited morphological similarities to the alveolar sacs in lungs. Subsequently, we established an *in vitro M*.*tb* infection model using hLOs (lung alveolar epithelium) and human macrophages (hMφs; immune cells) to more closely mimic the *in vivo* microenvironment. Additionally, we explored the potential of host-directed therapy (HDT) by regulating host genes using small interfering RNAs (siRNAs) to effectively treat intracellular *M*.*tb* in hLOs.

## Results

### Generation of hPSC-derived 3D lung organoids

**Fig 1:**
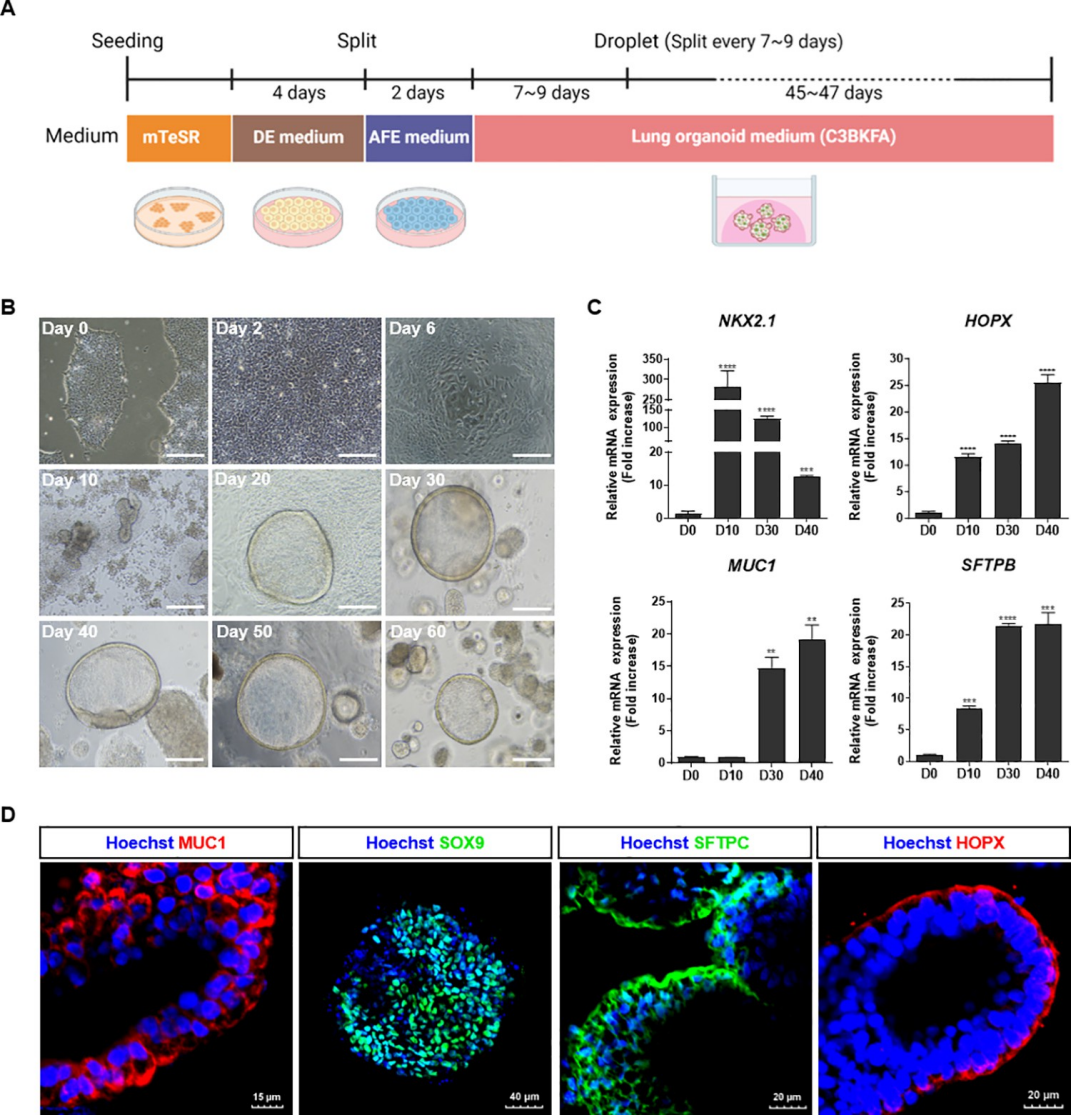
Generation and characterization of 3D hLOs derived from hPSCs. (A) Schematic diagram of generation of hPSC-derived LOs. Figure Created with BioRender.com. (B) Microscopy images showing the morphological changes from hPSCs (day 0) to endoderm (day 2), anterior foregut (day 6), and then to LOs over time. Scale bar, 200 μm. (C) Relative mRNA expression levels of lung specific markers (NKX2.1, HOPX, MUC1, and SFTPB). Groups were compared using a one-way analysis of variance followed by Dunnett’s multiple comparisons test. ****p < 0.0001, ***p < 0.001, and *p < 0.05. The experiments were repeated at least three times. (D) Immunofluorescence images showing the expression of mucous cell marker MUC1 (red), distal cell marker SOX9 (green), alveolar type 2 cell marker SFTPC (green), and alveolar type 1 cell marker HOPX (red) in hLOs.

To assess the long-term efficacy of the anti-TB drugs, we generated 3D lung organoids derived from hPSCs following a previously established protocol (Fig 1A) [18–20]. Briefly, hPSCs were differentiated into definitive endodermal (DE) cells, which were then re-plated onto Matrigel-coated plates and cultured in an anterior foregut endoderm (AFE) differentiation medium. Following AFE differentiation, the cells were embedded in Matrigel droplets and cultured for an additional 60 days, with passages every 7–9 days in LO medium to establish hLOs.

hLOs were successfully formed and exhibited a structure with a hollow lumen similar to that of the alveolar sacs (Fig 1B). Real-time PCR (qPCR) data showed that the expression of lung progenitor cell marker NKX2.1 gradually decreased, while that of mucus-secreting cell (MUC1), alveolar type 1 epithelial cell (AEC1; HOPX), and alveolar type 2 epithelial cell (AEC2; SFTPB) markers gradually increased as hPSCs differentiated into hLOs (Fig 1C). Immunofluorescence staining also showed the expression of distal lung markers in hLOs, such as MUC1, SOX9, SFTPC, and HOPX [21–23], confirming the successful generation of multicellular 3D hLOs containing mucus-secreting cells, lung progenitor cells, AEC1, and AEC2 from hPSCs (Fig 1D). Furthermore, we found that small airway epithelial cell markers (MUC5AC, goblet cell; SCGB1A1, club cell; p63, basal cell) were expressed in a small proportion of hLOs, indicating that they are mainly composed of alveolar type cells (S1 Fig).

### Establishment of an *M*.*tb* infection model using hLOs containing hMφs

**Fig 2:**
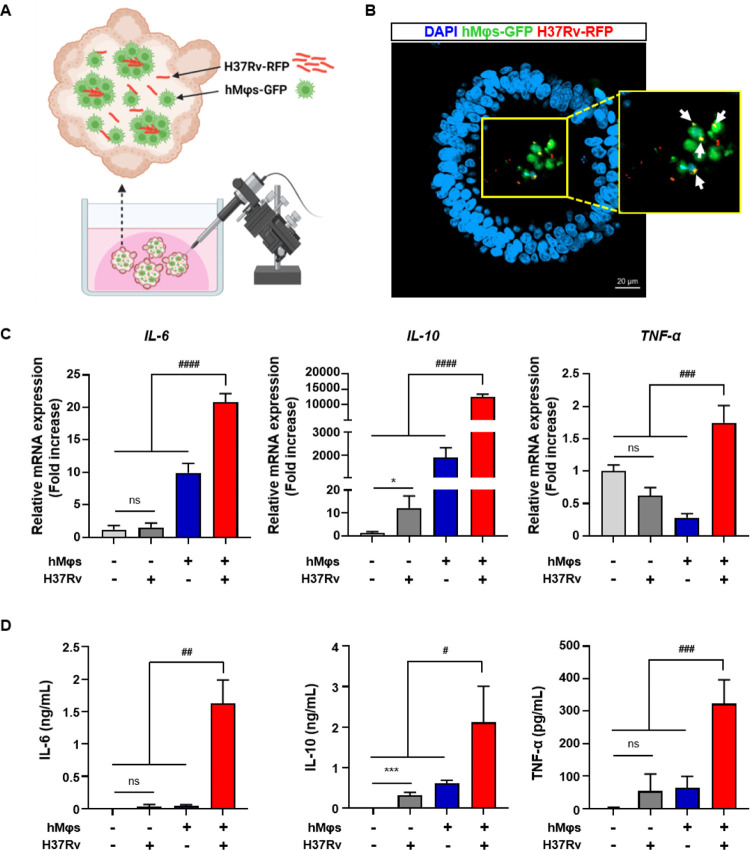
Establishment of an *M*.*tb* infection model using hPSC-derived hLOs containing hMφs. (A) Scheme of construction of an *M*.*tb* H37Rv infection model. Figure Created with BioRender.com. (B) Fluorescence images of hMφs-GFP and H37Rv-RFP in hLOs. hMφs-GFP and *M*.*tb* H37Rv were micro-injected into hLOs and incubated for 6 h. The yellow box indicates the magnified region. Scale bar, 20 μm. (C) Relative mRNA and (D) protein expression levels of cytokines (IL-6, IL-10, and TNF-α) in hLOs containing hMφs infected with *M*.*tb* H37Rv for 48 h. hLO alone and *M*.*tb*-infected hLO (without macrophages) were set as negative controls. The experiments were repeated at least three times. Statistically significant differences were determined using an unpaired two-tailed *t*-test. *p<0.05 and ***p<0.001. ^#^*p*<0.05, ^##^*p*<0.01, ^###^*p*<0.001 and ^####^*p*<0.0001.

**Fig 3:**
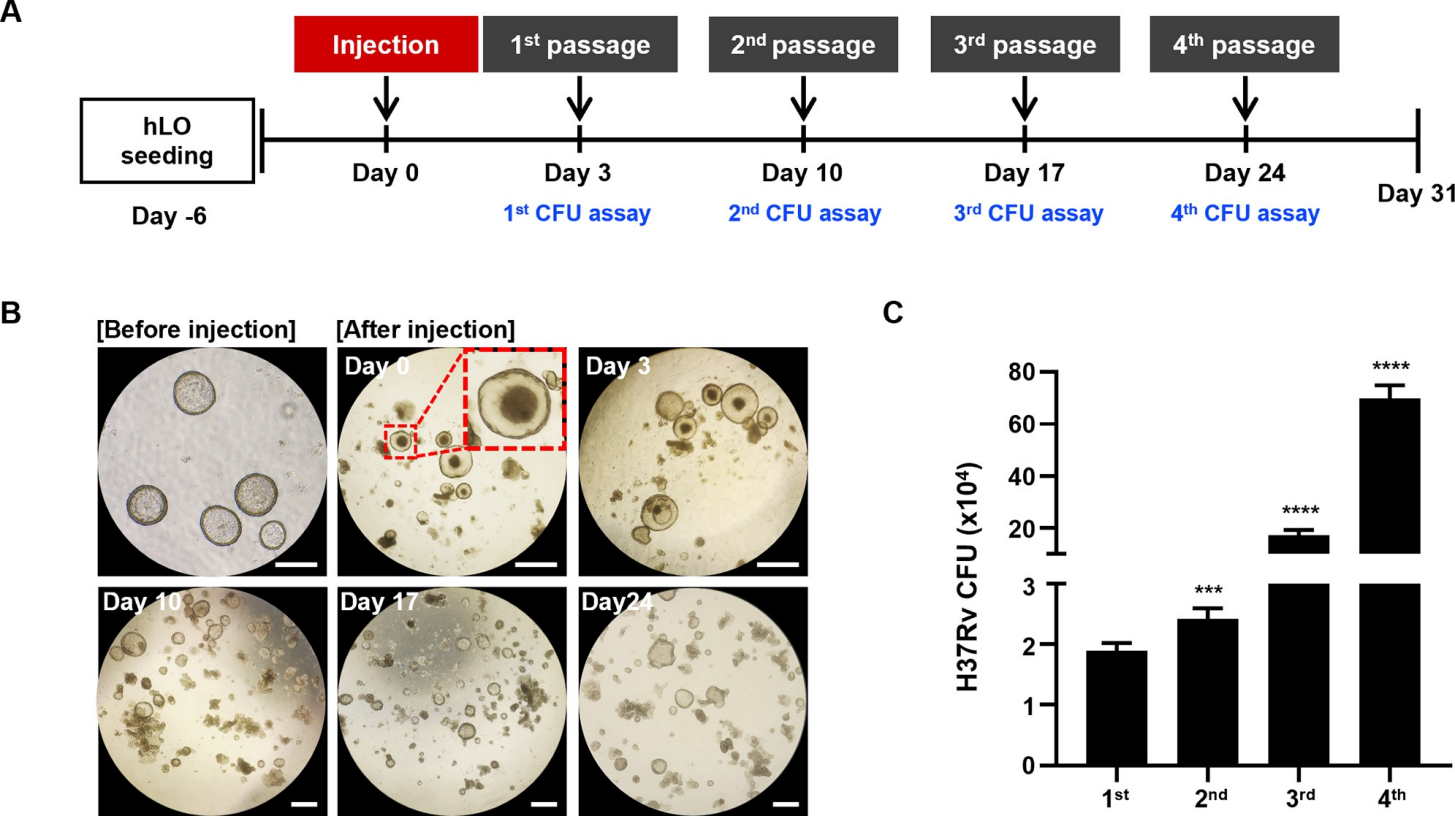
Establishment of a long-lived *M*.*tb* infection model using hLOs. (A) Schematic of the experimental time course. hMφs and H37Rv were micro-injected after 6 days of hLO seeding. CFU assays were performed on days 3, 10, 17, and 24 post-infection, and hLOs were passaged every 7 days at a 1:6 ratio. (B) Microscopy images of hLOs micro-injected with hMφs and H37Rv. A magnified image of the red box indicates the existence of hMφs and *M*.*tb* H37Rv in hLOs. Scale bar, 200 μm. (C) Determination of *M*.*tb* H37Rv viability in hLOs via CFU assay after 7 days of each passage. The experiments were repeated at least three times. Statistically significant differences were determined using an unpaired two-tailed *t*-test. ****p < 0.0001 and ***p < 0.001.

As shown in Fig 1 and 3D hLOs comprising human lung ACEs were successfully established. To replicate the *M*.*tb*-infected lung tissue environment, we aimed to create a model that enables structural interactions between macrophages and *M*.*tb* within the hLO lumen. Our previous study [19], confirmed the presence of macrophages in hLOs. However, owing to the low proportion of macrophages for verifying immune response, we micro-injected macrophages into hLOs to achieve an appropriate proportion. Initially, we differentiated green fluorescent protein (GFP)-expressing human monocytes into hMφs with phorbol myristate acetate (PMA)/Ionomycin (S2A Fig). FACS analysis revealed robust expression of macrophage-specific markers CD14, CD209, CD206, CD11b, CD36, and CD68 (S2B Fig) [24, 25]. In addition, cell aggregation indicating phagocytosis was observed under a fluorescence microscope, and there was an increase in gene expression and secretion of pro- or anti-inflammatory cytokines following infection of hMφs-GFP with H37Ra-red fluorescent protein (RFP) (S2C–S2E Fig). H37Rv-RFP and hMφs-GFP were micro-injected into the established hLO lumen (Fig 2A and S1 Video), and interestingly, H37Rv-RFP was observed to be surrounded by hMφs-GFP in the hLO lumen within 6 h after micro-injection (Fig 2B). Subsequently, we examined the immune response of hMφs-GFP-containing hLOs to H37Rv-RFP infection via qPCR analysis and enzyme-linked immunosorbent assay (ELISA). The results indicated a significant increase in the mRNA and protein expression levels of the inflammatory cytokines IL-6, IL-10, and TNF-α 48 h after H37Rv-RFP infection compared with levels in the controls (hLO alone, *M*.*tb*-infected hLO, and hLO containing hMφs) (Fig 2C and 2D). These results suggest the successful generation of an *M*.*tb* infection model using hLOs containing hMφs.

### Long-term culture of *M*.*tb*-infected hLO model

Owing to their short survival period, current *in vitro* models of *M*.*tb* infection using hMφ cell lines have limitations as preclinical drug efficacy evaluation models. These results prompted us to develop a new long-lived infection model for assessing the efficacy of anti-TB drugs in the treatment of latent TB infection. To address the fate of *M*.*tb*-infected hMφs, we have measured cell cytotoxicity and viability of hMφs after H37Rv infection (MOI = 1) for 96 hours. As a result, hMφs died within 72–96 h after *M*.*tb* infection (S5A Fig), showing an increase in LDH release (cell cytotoxicity) and a decrease in cell viability (S5B and S5C Fig). These results support that hMφs phagocytize *M*.*tb* and die within 96 hours during intracellular *M*.*tb* replication, indicating the challenge of long-term culture. Therefore, we developed a long-term culture method and established a long-lived *M*.*tb* infection model using hLOs. On day 3 of *M*.*tb* infection, hLOs containing hMφs-GFP and H37Rv-RFP were passaged and embedded in growth factor-reduced (GFR) Matrigel mixed with LO medium in 24-well plates, after which H37Rv-RFP infected hLOs were passaged every 7 days (Fig 3A). The presence of hMφs-GFP and H37Rv-RFP in hLOs was observed after micro-injection, and hLOs proliferated over one month (Fig 3B). Colony-forming unit (CFU) assays were performed at every passage to estimate the viability of H37Rv-RFP in hLOs. The CFU values increased over 25-folds after three passages (Fig 3C). These findings support the successful development of an *M*.*tb* infection model that can last over one month and preserve the 3D morphological structure of hLOs.

### Assessment of anti-TB drug efficacy using the long-lived *M*.*tb* infection model

**Fig 4:**
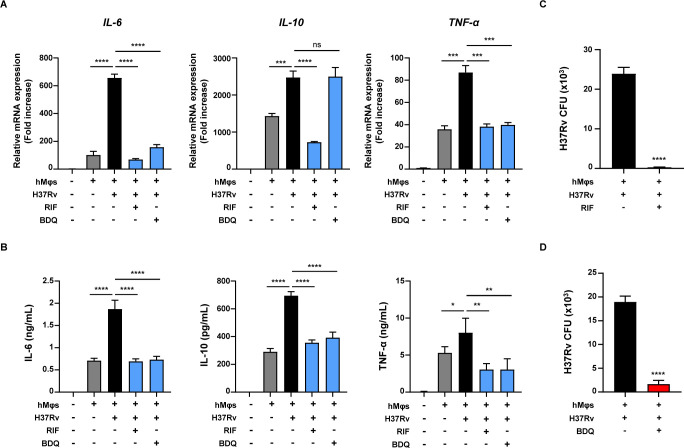
Assessment of the efficacy of anti-TB drugs using the *M*.*tb* infection model. The *M*.*tb* H37Rv infection model was treated with 20 μg/mL RIF or 5 μg/mL BDQ for 48 h. (A) Relative mRNA expression levels and (B) cytokine (IL-6, IL-10, and TNF-α) production in *M*.*tb* H37Rv-infected hLOs. (C, D) Intracellular survival of *M*.*tb* H37Rv in hLOs was assayed using CFU assay at 48 h post-treatment of anti-TB drugs. The experiments were repeated at least three times. Statistically significant differences were determined using an unpaired two-tailed *t*-test. *p<0.05, **p<0.01, ***p<0.001 and ****p<0.0001.

*In vitro* drug testing is crucial to predict drug efficacy in clinical trials. Therefore, it is necessary to determine whether our *M*.*tb* infection model is suitable for evaluating the efficacy of anti-TB drugs as a preclinical model. We treated the *M*.*tb* infection model with the anti-TB drugs rifampicin (RIF) and bedaquiline (BDQ) for 48 h (Fig 4). We observed that the mRNA expression level and protein production of cytokines such as IL-6, IL-10 and TNF-α increased upon *M*.*tb* H37Rv infection and significantly decreased following treatment with RIF and BDQ, respectively (Fig 4A and 4B). Additionally, treatment with RIF and BDQ effectively reduced intracellular *M*.*tb* survival by 98.6 and 91.3%, respectively (Fig 4C and 4D).

**Fig 5:**
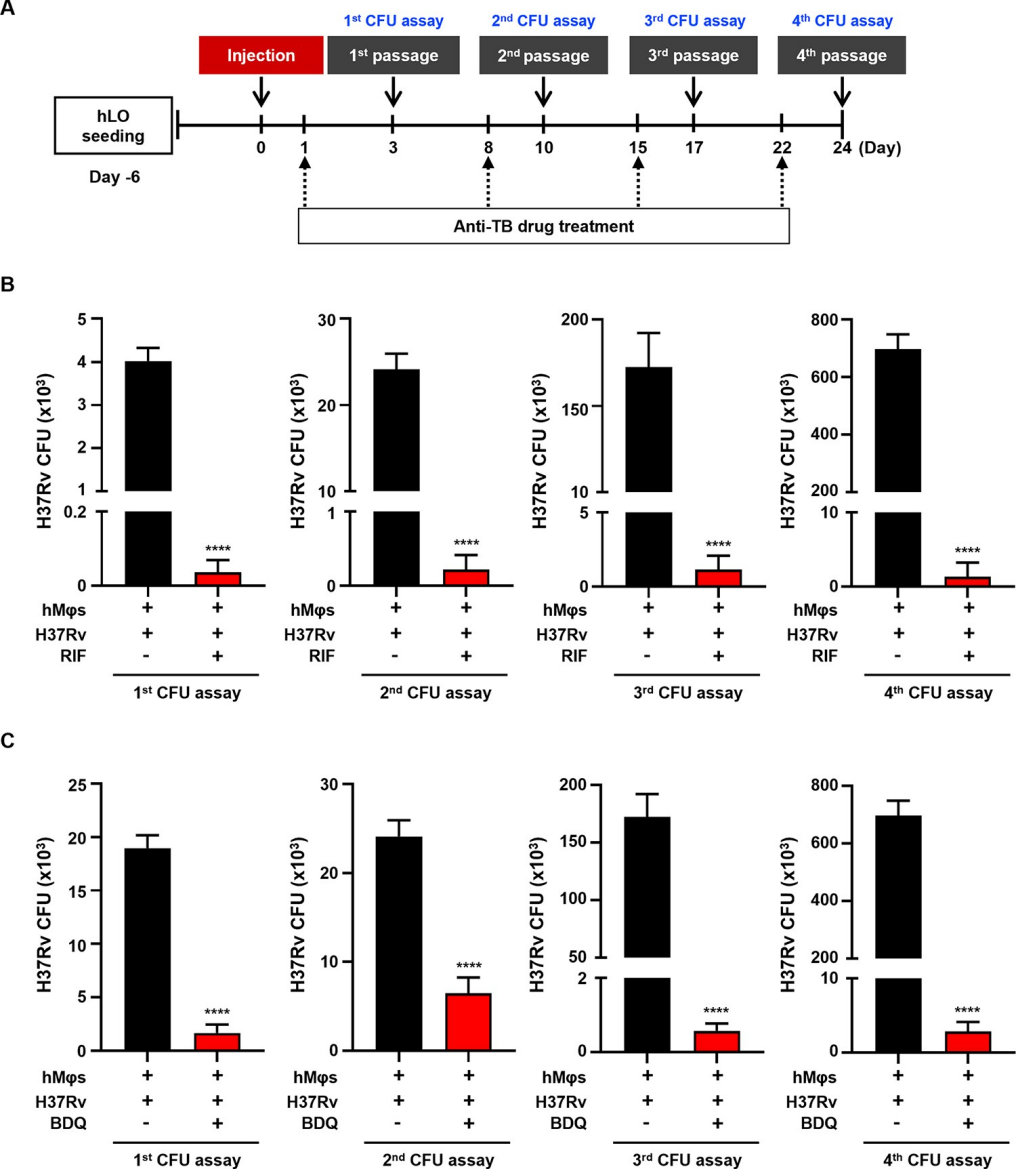
Assessment of anti-TB drug efficacy using the long-lived *M*.*tb* infection model. (A) Schematic diagram of experimental time course for treatment with anti-TB drugs in long-lived *M*.*tb* infection model. (B, C) Intracellular survival of *M*.*tb* H37Rv in hLOs was assayed using CFU assay at each passage followed by post-treatment with anti-TB drugs. *M*.*tb* H37Rv infection model was treated with (B) 20 μg/mL RIF and (C) 5 μg/mL BDQ for 48 h. The experiments were repeated at least three times. Statistically significant differences were determined using an unpaired two-tailed *t*-test. ****p < 0.0001.

To assess the suitability of our *M*.*tb* infection model for long-term efficacy testing, H37Rv-infected hLOs were passaged four times every 7 days and treated with RIF (20 μg/mL) and BDQ (5 μg/mL) by adding prepared dilution to the cell culture media for 48 h before CFU assay (Fig 5A). *M*.*tb* growth was evaluated via CFU assay at each passage, revealing that treatment with RIF and BDQ significantly reduced intracellular *M*.*tb* H37Rv survival. RIF decreased CFU values by more than 99%, that is, 99.1, 99.1, 99.3, and 99.8% at each passage, respectively (Fig 5B). Likewise, BDQ treatment reduced CFU values by 91.3, 73.1, 99.7, and 99.6% at each passage, respectively (Fig 5C). Similar observations were made with isoniazid (INH) treatment (97.3, 97.3, 99.6, and 99.4% reduction at each passage, respectively) (S3 Fig).

### Evaluation of the efficacy of HDT using the *M*.*tb* infection model

Next, the efficacy of HDT in removing intracellular *M*.*tb* was evaluated in the established hLOs. We have previously reported that the knockdown of HERPUD1 and MFN2 suppressed intracellular survival of *M*.*tb* in macrophages. Knockdown of MFN2 inhibited *M*.*tb* growth by disrupting the mitochondrial network, leading to apoptosis [26], while depletion of HERPUD1 resulted in increased ROS levels, leading to autophagy induction and decreased intracellular *M*.*tb* burden [27]. Therefore, we investigated the efficacy of HDT using HERPUD1 and MFN2 as target factors in TB treatment.

**Fig 6:**
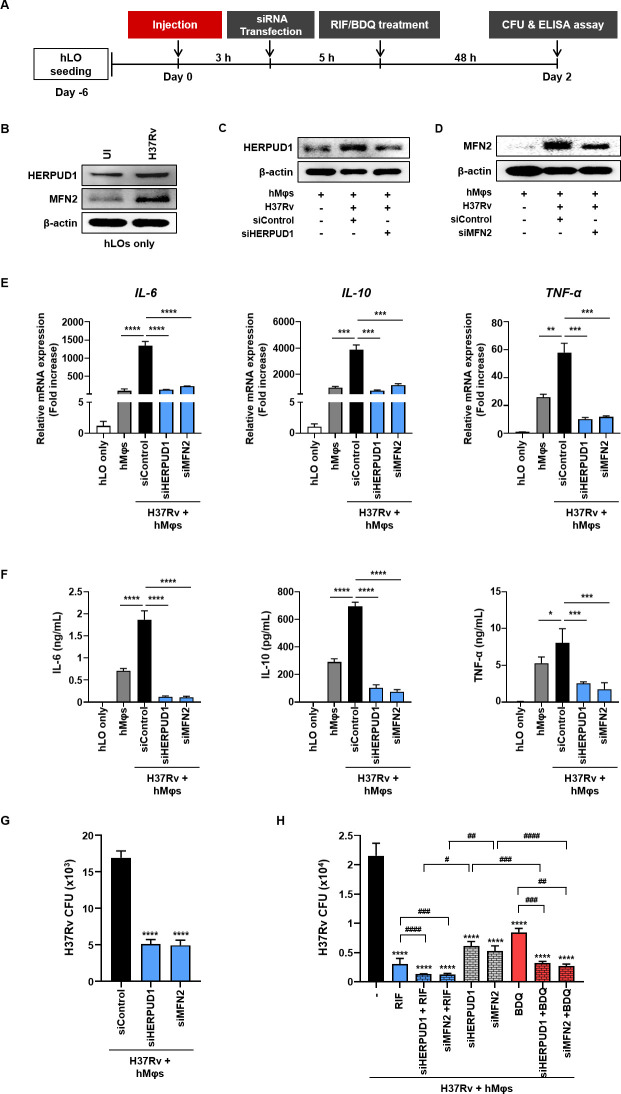
Evaluation of the efficacy of HDT using *M*.*tb* infection model. (A) Schematic diagram of the experimental time course for siRNA transfection in the *M*.*tb* infection model. (B) hLOs were infected with *M*.*tb* H37Rv for 48 h. The protein levels of HERPUD1 and MFN2 were determined using western blotting. UI, uninfected control. (C-F) *M*.*tb* H37Rv-infected hLOs were transfected with siRNA (siHERPUD1 or siMFN2, 200 nM) for 48 h. The protein levels of (C) HERPUD1 and (D) MFN2 were determined at 48 h using western blotting. (E) The mRNA level and (F) protein production of inflammatory cytokines at 48 h. (G) Intracellular survival of *M*.*tb* H37Rv in hLOs was assayed at 48 h using CFU analysis. The experiments were repeated at least three times. Statistically significant differences were determined using an unpaired two-tailed t test. ****p<0.0001, ***p<0.001, **p<0.01 and *p<0.05. (H) *M*.*tb* H37Rv-infected hLOs were transfected with siRNA (siMFN2 or siHERPUD1, 200 nM) and then treated with 1 μg/mL RIF or 0.1 μg/mL BDQ for 48 h. Intracellular survival of *M*.*tb* H37Rv in hLOs was assayed at 48 h using CFU analysis. The experiments were repeated at least three times. Significant differences from the H37Rv control are indicated by asterisks (*) (****p<0.0001). Significant differences from the control (anti-TB drugs or siRNA) are indicated by asterisks (^#^) (^#^*p*<0.05; ^##^*p*<0.01 and ^###^*p*<0.001, ^####^*p*<0.0001).

Initially, we observed induction of protein expression for HERPUD1 and MFN2 in hLOs (without macrophages) and BEAS-2B cells, human epithelial cell line by *M*.*tb* infection (Fig 6B and S4C). Additionally, *M*.*tb* exhibited robust survival in BEAS-2B cells and hLOs (without macrophages) (S4A and S4B Fig). We transfected *M*.*tb* H37Rv-infected hLOs with specific siRNAs targeting MFN2 or HERPUD1 for 48 h, effectively reducing the protein expression of HERPUD1 and MFN2 (Fig 6C and 6D). Subsequently, we observed that the mRNA expression levels and protein production of cytokines such as IL-6, IL-10 and TNF-α increased upon *M*.*tb* H37Rv infection and significantly decreased following treatment with specific siRNA, respectively (Fig 6E and 6F). Moreover, the knockdown of MFN2 or HERPUD1 significantly reduced the intracellular survival of *M*.*tb* (Fig 6G). Similar results were observed in BEAS-2B cells transfected with siRNA against HERPUD1 and MFN2 (S4D–S4G Fig). Furthermore, we evaluated the intracellular number of *M*.*tb* after combination therapy with anti-TB drugs and siRNAs. As expected, the intracellular survival of *M*.*tb* significantly decreased when combination therapy was administered compared to that of the control group (anti-TB drug treatment alone and siRNA transfection alone) (Fig 6H). Although these findings represent the initial trials of HDT in hLOs, they suggest that our infection model with PSC-derived hLOs and monocyte-derived hMφs holds promise as a method for anti-TB drug testing and HDT approaches.

## Discussion

As *M*.*tb* infection progresses, continuous recruitment and aggregation of immune cells, including macrophages, leads to granuloma formation [2,28]. Although *M*.*tb* can infect and replicate within alveolar epithelial type II cells [29–31] and alveolar macrophages are important as early predators, the recruited monocytes and monocyte-derived macrophages play a protective role during *M*.*tb* infection [32]. Considering the significant role of macrophages during infection, in this study, we used PMA-differentiated THP-1 cells as hMφs to establish hLOs. After H37Ra-RFP infection, hMφs expressed inflammatory cytokine genes, and hMφs surrounding macrophages infected with *M*.*tb* H37Ra-RFP were observed (S2 Fig). In this study, we found that *M*.*tb*-infected hLOs were maintained after several passages. Moreover, the intracellular growth of *M*.*tb* during the long-term culture of hLOs was predicted due to the invasion of *M*.*tb* into epithelial cells and its survival inside the epithelial cells over a few passages. It seems that epithelial cells in hLOs support *M*.*tb* replication all along the 4th passages, since hMφs died within 96 hours after *M*.*tb* infection based on S5 Fig. Therefore, in the early time of infection, macrophages phagocytize *M*.*tb* and die within 96 hours during intracellular *M*.*tb* replication. When macrophages die, *M*.*tb* infection spreads to epithelial cells and replicates in the cells of hLOs.

Considering the challenges in TB modeling to accurately replicate the *in vivo* microenvironment, it is essential to co-culture epithelial and immune cells. For modeling *M*.*tb* infection, a lung-on-chip model involving co-culture of mouse epithelial cells, endothelial cells, and Mφs has been employed [13]; however, this model lacks a 3D structure that can replicate the *in vivo* lung environment. Another study attempted to co-culture human monocyte-derived macrophages with human adult stem cell-derived airway organoids (hAOs) containing *Mycobacterium bovis* BCG [14]. Nonetheless, this co-culture system also fails to mimic the *in vivo* environment, as macrophages cannot traverse the basal side to access the lumen of hAOs and remove BCG. Here, we demonstrated that micro-injecting H37Rv-RFP and hMφs-GFP into the lumen of hLOs may be more efficient for investigating pathogen-host interactions in a lung-like environment.

*In vivo* granulomas involves a range of immune cells, including dendritic cells, T cells, B cells, and macrophages [2]. Given the intricacies of recreating diverse *in vivo* microenvironments, most granulomas studies have relied on tissue biopsies or *in vivo* animal models [33,34]. *In vitro* models present significant challenges owing to the complexity of the environment. Our system also has limitations in that hLOs need to encompass macrophages, and other types of immune cells to accurately replicate granuloma formation.

As organoids consist of complex and diverse variables, including cell state and cell type, achieving uniform organoid production is crucial for reducing variation and enhancing standardization. In this study, we employed a cell counting-based approach to seed hLOs and analyzed each well as a single reaction, thereby minimizing the variability between wells. The organoid production method, which relies on cell counting, effectively minimizes variations among wells, rendering it suitable for the comparative evaluation of the efficacy of anti-TB drug testing.

The *M*.*tb* infection hLO model we constructed offers several advantages over the infection models presented in previous studies [14,35]. First, our hLO-based *M*.*tb* infection model maintained its 3D shape, even after a single infection, for at least four passages during long-term culture. Second, the number of *M*.*tb* within the hLOs remained consistent after long-term passage, indicating the suitability of this model for drug testing. Although another study demonstrated the long-term survival of *Mycobacterium abscessus* (*M*.*ab*) in hAOs for 21 days [14], their hAO infection model was less suitable for drug testing due to the absence of passaged cultures of hAOs infected with *M*.*ab*, unlike our infection model. Since the long-lived *M*.*tb* infection model presented in this study can be maintained for up to 31 days, it is possible to analyze the anti-TB effect of new anti-TB drugs and determine whether anti-TB drugs can solve the problem of *M*.*tb* relapse. Third, our *M*.*tb* infection model can produce a minimum of 216 daughter hLOs, each containing a similar number of *M*.*tb*, after four passages during long-term culture with a single infection. We observed that treatment with anti-TB drugs significantly reduced intracellular *M*.*tb* survival and *M*.*tb-*induced inflammatory cytokine levels in hLOs. *M*.*tb* stimulation induces the production of inflammatory cytokines, which are a marker of disease activity and inflammation in TB [36]. Measurement of inflammatory cytokines is crucial as it serves as an indicator by which treatment efficiency can be assessed [37,38].

Our infection system is a promising method for evaluating the efficacy of novel drugs and HDT. Currently, numerous HDT approaches are available for TB therapy, including enhancing autophagy, promoting phagosome maturation, inhibiting mTOR, inhibiting inflammation or necrotic cell death, and cytokine therapy [39]. However, most of these approaches have been investigated in two-dimensional cell models, mice, and clinical trials. In this study, we demonstrated the use of hLOs as a model for drug testing against *M*.*tb* infection using several host-specific siRNAs targeting HDT candidates, such as MFN2 and HERPUD1 [26,40]. We have previously reported that the knockdown of MFN2 inhibited the growth of *M*.*tb* by disrupting the mitochondrial network, leading to apoptosis [26] and reduction of intracellular *M*.*tb*. Previously, we reported that the knockdown of HERPUD1 also contributes to in reducing the number of *M*.*tb* by increasing ROS-mediated autophagy within macrophages [27]. Consistent with our previous findings, the knockdown of HERPUD1 and MFN2 inhibited intracellular *M*.*tb* growth and reduced the production of inflammatory cytokines. This reduction in cytokines production by HDT and anti-TB drug treatment in the *M*.*tb*-infected hLO model can be considered as an indicator of the reduction of intracellular *M*.*tb* survival in host cells. Our findings are consistent with previous reports showing decreased inflammatory cytokines levels during successful TB cure with anti-TB drugs [37,38,41]. Additionally, HDT strategies offer dual advantages in controlling TB by enhancing the efficacy of anti-TB drugs and by reducing tissue inflammation and disease pathology [42,43].

These findings suggest that the *M*.*tb* infection model we developed using PSC-derived hLOs and monocyte-derived hMφs is useful for evaluating TB treatment using HDT. In conclusion, *M*.*tb* H37Rv can grow in hPSC-derived hLOs for at least 4 weeks, making our *M*.*tb* infection model suitable for testing the efficacy of anti-TB drugs and HDT that require a prolonged therapy period.

## Materials and methods

### Ethics statement

H9 cell line was obtained from the WiCell Research Institute (Madison, WI, USA). This study was approved by the Public Institutional Review Board of Ministry of Health and Welfare (Approval number: #P01-202104-41-001).

### Maintenance of human pluripotent stem cells

hPSCs were maintained as follows: H9 cells were cultured in mTeSR1 (STEMCELL Technologies, Vancouver, Canada) on hESC-qualified Matrigel (Corning Inc., Corning, NY, USA)-coated tissue culture plates (Thermo Fisher Scientific, Waltham, MA, USA) in a humidified 5% CO_2_ incubator at 37°C. H9 cells were passaged every 3–4 days using 0.5 mM ethylenediaminetetraacetic acid, and the medium was replaced with fresh mTeSR1 daily.

### Generation of human pluripotent stem cell-derived 3D lung organoids

hPSCs were differentiated into 3D hLOs as previously described [18]. Briefly, H9 cells were seeded onto hESC-qualified Matrigel-coated cell culture plates. After 3 days, they were differentiated into DE cells for 4 days using the STEMdiff Definitive Endoderm Kit according to the manufacturer’s protocol (STEMCELL Technologies). DE basal medium supplemented with MR and CJ was replaced daily, followed by 48 h of supplementation with CJ alone. Subsequently, the cells were split at a density of 1E5 cells/cm^2^ for AFE differentiation in a medium consisting of Dulbecco’s modified Eagle’s medium/F12 medium (DMEM/F12; Gibco, NY, USA) with B27 (1X; Gibco), N2 (1X; Gibco), penicillin-streptomycin (100 U/mL; Gibco), Noggin (100 ng/mL; Peprotech, Rocky Hill, NJ, USA), SB431542 (10 μM; Tocris, Bristol, UK), and IWP-4 (1 μM; Stemgent, Cambridge, MA, USA). After 2 days, the medium was changed to DMEM/F12-based LO medium containing B27, N2, penicillin-streptomycin, BMP4 (10 ng/mL; Peprotech), FGF10 (10 ng/mL; Peprotech), KGF (10 ng/mL; Peprotech), all-trans retinoic acid (50 nM; R&D Systems, Minneapolis, MN, USA), and CHIR99021 (3 μM; Tocris) for 7–9 days. Cell clumps were then harvested by mechanically pipetting up and down, embedded in GFR Matrigel (Corning Inc.) mixed with LO medium (3:2 ratio), and cultured in 24-well plates (50 μL droplet/well) in LO medium. The medium was replaced every 2–3 days. The hLOs were passaged every 7–9 days owing to the destruction of the Matrigel drop structure, which supports organoids as an extra cellular matrix. First, hLOs were harvested from Matrigel by pipetting and centrifugation. Next, hLOs were then broken into small fragments by mechanically pipetting up and down. Finally, hLOs were embedded in GFR Matrigel (Corning Inc.) mixed with LO medium (3:2 ratio) in 24-well plates (50 μL droplet/well) at a split ratio of 1:6–1:12 and the medium was replaced every 2–3 days. Organoids between 50 and 60 days of differentiation were used in the experiments.

### Differentiation of human monocyte-derived macrophages

The human monocytic leukemia cell line (THP-1 cells; ATCC, Manassas, VA, USA) was used as human monocytes and was maintained in Roswell Park Memorial Institute 1640 medium (Gibco) supplemented with fetal bovine serum (10%; Gibco), β-mercaptoethanol (0.05 mM; Gibco), HEPES (10 mM; Gibco), sodium pyruvate (1 mM; Gibco), and penicillin-streptomycin (100 U/mL; Gibco) in a humidified 5% CO_2_ incubator at 37°C. To visualize THP-1 cells co-cultured with hLOs, they were transduced with a retroviral GFP vector, and GFP^+^-sorted cells were used in the experiments. For differentiation into hMφs, THP-1-GFP cells were seeded at a density of 1E5 cells/cm^2^, stimulated with a cell activation cocktail (PMA and Ionomycin; BioLegend, San Diego, CA, USA), and incubated for 18–24 h (S1A Fig). Upon observation of adherence, THP-1-derived hMφs were harvested using Cell Dissociation reagent (STEMCELL Technologies) and used in the experiments.

### qPCR

Cells were collected, washed with Dulbecco’s phosphate-buffered saline (DPBS; WELGENE, Korea), and lysed with TRIzol reagent (Invitrogen, Carlsbad, CA, USA). Total RNA was extracted via the TRIzol/chloroform/isopropanol precipitation method and the RNA was reverse transcribed into cDNA using GoScript Reverse Transcriptase (Promega, Madison, WI, USA). The mRNA expression of lung-specific genes (NKX2.1, HOPX, MUC1, and SFTPB), and proinflammatory cytokine-related genes (IL-6, IL-10, TNF-α, MCP-1, IL-1α, and IL-1β) was measured on a StepOnePlus Real-Time PCR system (Applied Biosystems, Foster City, CA, USA) using GoTaq qPCR Master Mix (Promega). The primers used for qPCR are listed in the S1 Table.

### ELISA

Secretion of TNF-α (BD bioscience), IL-6 (BD bioscience), and IL-10 (R&D system) in the medium from cultured *M*.*tb*-infected hLOs was determined using sandwich ELISA. All assays were performed according to the manufacturer’s instructions. Triplicate samples were analyzed using SpectraMax ABS Microplate Reader (Molecular Devices, CA, USA) and compared to a standard curve.

### Transfection of small interfering RNA

hLOs were seeded 6 days before micro-injection with *M*.*tb* H37RV and macrophages. *M*.*tb* H37Rv and hMφs were micro-injected into hLOs, and 3 h after micro-injection, *M*.*tb* H37Rv-infected-hLOs were transfected with siRNA for 5 h. Thereafter, the medium was replaced and then hLOs were cultured in fresh medium. siControl (200 nM; Bioneer, Daejeon, South Korea), siMFN2 (200 nM; Bioneer), and siHERPUD1 (200 nM; Bioneer) were transfected into hLOs using Lipofectamine 3000 (Invitrogen) according to the manufacturer’s instructions.

### Immunofluorescence

hLOs were harvested, washed with PBS, and then fixed in 4% paraformaldehyde for 1 h at 4°C. After washing twice with PBST (0.1% Tween 20 in PBS), hLOs were permeabilized with 0.5% Triton X-100 solution for 15 min at 25°C and blocked with blocking buffer (2% bovine serum albumin and 0.1% Triton X-100 in PBS). Subsequently, hLOs were incubated with primary antibodies (diluted in blocking buffer) against MUC1 (1:50; Santa Cruz Biotechnology Inc., Dallas, TX, USA), SOX9 (1:200; Abcam, Cambridge, UK), SFTPC (1:50; Invitrogen), and HOPX (1:50; Santa Cruz Biotechnology Inc.) in blocking buffer overnight at 4°C. Excess primary antibodies were removed by washing the hLOs thrice with PBST, followed by incubation with the fluorescence-conjugated secondary antibodies Alexa Fluor 488-conjugated anti-rabbit IgG and 594-conjugated anti-mouse IgG (diluted 1:1,000 in blocking buffer, Invitrogen) for 2 h in the dark at room temperature. The primary and secondary antibodies used are listed in the S2 Table. hLOs were washed thrice with PBST to remove excess antibodies. Nuclei were stained with Hoechst 33342 (1:1,000; Thermo Fisher Scientific). hLOs were imaged using an FV3000 confocal microscope (Olympus, Tokyo, Japan), and images were analyzed using FV31S-SW Viewer software (version 2.3.2.169; Olympus).

### Bacterial culture and CFU assay

The *M*.*tb* strain H37Rv (ATCC 27294) was purchased from the American Type Culture Collection (Manassas, VA, USA) and cultured in Middlebrook 7H9 liquid medium containing 10% oleic acid, albumin, dextrose, and catalase combined with 5% glycerol [28]. Aliquots of the upper bacterial suspension of *M*.*tb* H37Rv were frozen at −80°C until use. hLOs were infected with *M*.*tb* H37Rv at a multiplicity of infection (MOI) of 1 for 48 h. hLOs were lysed in autoclaved distilled water to release the intracellular bacteria, after which the lysates were plated separately on 7H10 agar plates and incubated at 37°C for 21 days. Colony counts were performed in triplicate.

### *M*.*tb* infection model

On days 50–60 of differentiation, hLOs were harvested and mechanically dissociated into small fragments by pipetting. The hLOs were then split at a density of 2.0 × 10^4^ cells, embedded in GFR Matrigel mixed with LO medium (3:2 ratio) in four-well plates (50 μL droplet/well), and maintained in antibiotic-free LO medium. On the day of micro-injection, cells per well were counted several times by dissociating hLOs into single cells using TrypLE. Based on cell counting, approximately 1–2 × 10^5^ hMφs-GFP and H37Rv-RFP (MOI = 1) were micro-injected into hLOs at the same density as hLOs per well, which is similar to the ratio of lung epithelial cells and macrophages in the human lungs [44] (hLO:hMφs-GFP:H37Rv-RFP = 1:1:1; Fig 2A and S1 Video). After 6–12 h, *M*.*tb* infection models were treated with anti-TB drugs for 48 h and then used in the experiments. For the long-term culture of the *M*.*tb*-infected hLO model, hLOs were passaged using the same procedures for hLOs. The hLOs at each passage contained a similar number of *M.tb* H37Rv.

### Cell viability assay

LDH release was measured by LDH cytotoxicity detection kit (Takara, Kusatsu, Japan) in the medium from cultured *M*.*tb*-infected THP-1 cells using a microplate reader (SpectraMax ABS). Cell viability was measured in *M*.*tb*-infected THP-1 cells by Cell Counting Kit-8 assay (CCK-8; Dojindo, Kumamoto, Japan) using a microplate reader.

### Statistical analysis

All data are presented as mean ± standard error of the mean. Statistical analyses were performed using GraphPad Prism software (version 9.5.1; GraphPad, Inc., San Diego, CA, USA). Statistical significance was assessed using a two-tailed unpaired *t*-test (*p* < 0.05). All experiments included at least three biologically independent replicates.

## Supporting information

S1 Fig Analysis of airway markers in hPSC-derived hLOs. (A) Relative mRNA expression of airway markers (P63, MUC5AC and SCGB1A1). Groups were compared using one-way analysis of variance followed by Dunnett’s multiple comparisons test. *p<0.05, **p<0.01 and ****p<0.0001. The experiments were repeated at least three times. (B) Immunofluorescence images showing the expression of basal cell marker P63 (green), goblet cell marker MUC5AC (red), and club cell marker SCGB1A1 (green) in hLOs.
(TIF)

S2 Fig Characterization of human monocyte-derived macrophages. (A) GFP-expressing human monocytes were activated by PMA/Ionomycin cocktail to induce monocyte-derived Mφs for 24 h. Representative images show changes in the morphology of monocytes. Figure Created with BioRender.com. (B) Some macrophage-specific markers (CD14, CD68, CD11b, Cd209, CD206, and CD36) were determined by flow cytometry after activation. Grey histograms represent isotype control. (C) Fluorescence microscopy images of induced cell aggregation in *M*.*tb* H37Ra*-*infected Mφs. (D) Relative mRNA expression levels of cytokines (IL-6, IL-10, TNF-α, MCP-1, IL-1α, and IL-1β) and (E) quantification of cytokine protein expression (IL-10 and TNF-α) produced by *M*.*tb* H37Ra*-*infected Mφs (MOI = 5). The experiments were repeated at least three times. Statistically significant differences were determined using an unpaired two-tailed *t*-test. ****p < 0.0001. Scale bars, 50 μm.
(TIF)

S3 Fig Efficacy of the anti-TB drug (INH) using the long-lived *M*.*tb* infection model. Intracellular survival of *M*.*tb* H37Rv in hLOs using CFU assay at each passage followed by post-treatment with anti-TB drug (INH). *M*.*tb* H37Rv infection model was treated with 10 μg/mL INH for 48 h before CFU assay. The experiments were repeated at least three times. Statistically significant differences were determined using unpaired two-tailed *t*-test. ****p < 0.0001.
(TIF)

S4 Fig *In vitro* infection of human epithelial cells with *M*.*tb* H37Rv. (A) Human epithelial cell line, BEAS-2B cells were infected with *M*.*tb* H37Rv (MOI = 1) for 24 h. Intracellular survival of *M*.*tb* H37Rv was assayed by CFU analysis. (B) Single-parameter flow cytometry histogram of RFP. hLOs were infected with *M*.*tb* H37Rv (MOI = 1) for 5 days. Upper panel shows gating strategy for flow cytometry analysis and lower panel shows the viability of *M*.*tb* H37Rv inside hLOs was detected by flow cytometry. Cells were selected from a FSC-A and SSC-A dot plot, and then gated in a FSC-A and FSC-H dot plot to eliminate doublets. Singlet cells were further analyzed for the expression of H37Rv-RFP to estimate *M*.*tb* growth. FSC-A, forward scatter area; SSC-A, side scatter area; FSC-H, forward scatter height. (C) Protein level of HERPUD1 and MFN2 was detected by western blot. UI, uninfected control. (D-G) BEAS-2B cells were transfected with specific siRNA for HERPUD1 or MFN2 (200 nM) and then infected with *M*.*tb* H37Rv (MOI = 1) for 48 h. Protein level of HERPUD1 or MFN2 and intracellular survival of *M*.*tb* H37Rv were analysis at 48 h after *M*.*tb* H37Rv infection. The experiments were repeated at least three times. Statistically significant differences were determined using unpaired two-tailed *t*-test. *p<0.05 and **p<0.01.
(TIF)

S5 Fig *In vitro* infection of hMφs with *M*.*tb* H37Rv. Human monocyte-derived macrophages (THP-1 cells) were infected with *M*.*tb* H37Rv (MOI = 1). Cell viability under *M*.*tb* infection was routinely monitored for 96 h through (A) fluorescence microscopy, (B) LDH assay, and (C) CCK-8 assay. The experiments were repeated at least three times. Statistically significant differences were determined using an unpaired two-tailed *t*-test. ****p<0.0001, ***p<0.001 and **p<0.01. Scale bar, 100 μm. UI, uninfected control.
(TIF)

S1 Table Primer sequences used for RT-qPCR. (TIF)

S2 Table Primary and secondary antibodies used for immunofluorescence. (TIF)

S1 Video Micro-injection of hMφs and *M*.*tb* H37Rv into hLOs. (MP4)

S1 Data The value for graphs. (XLSX)

S2 Data Western blot raw and uncropped images. (PDF)
