# Supplementary figures and images for: Advances in an In Vitro Tuberculosis Infection Model Using Human Lung Organoids for Host-Directed Therapies
Source: PLoS Pathog. 2024 Jul 25;20(7):e1012295. doi: 10.1371/journal.ppat.1012295 (PMC11271890; doi:10.1371/journal.ppat.1012295)

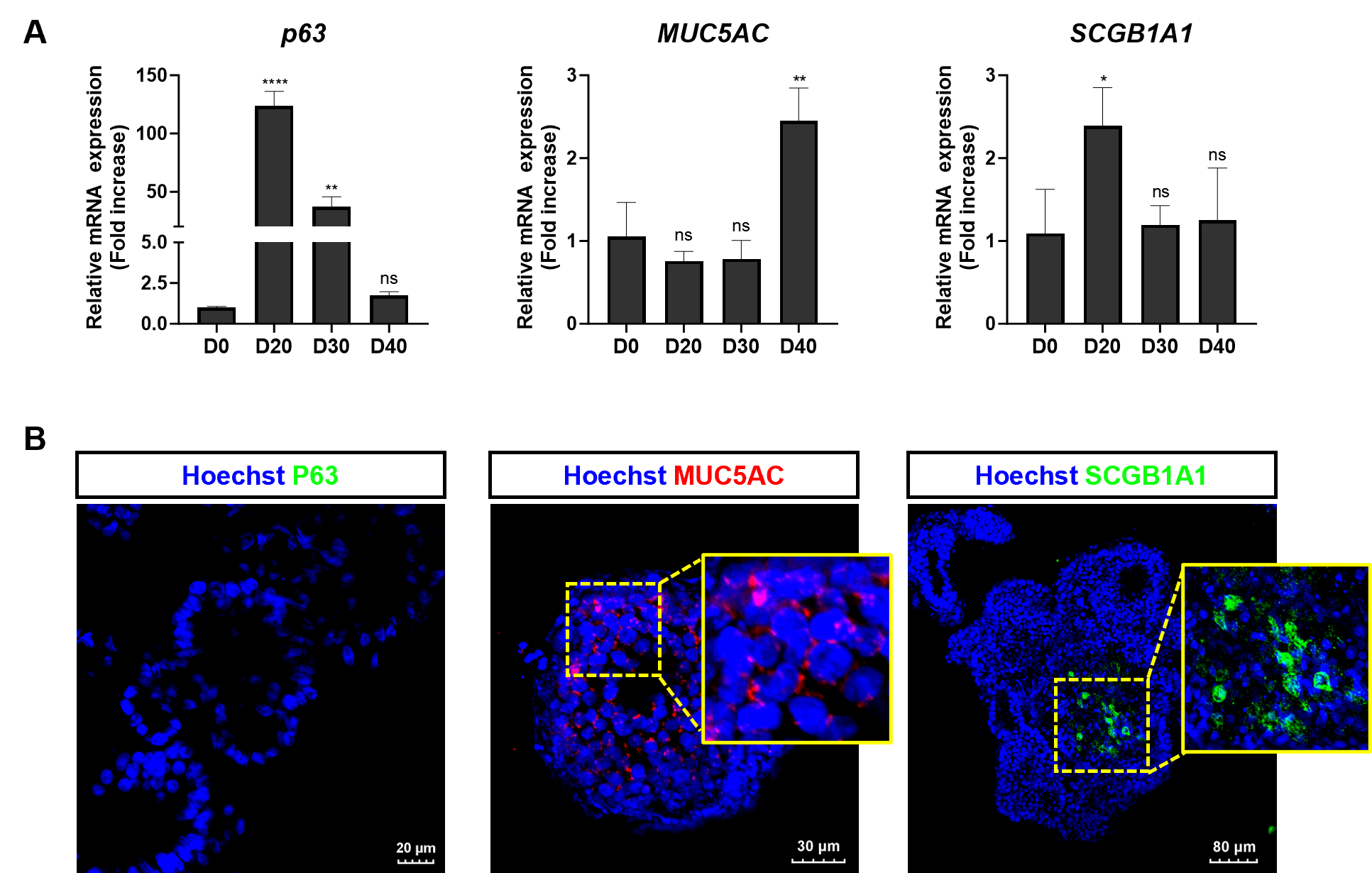

Supplement: S1 Fig — (A) Relative mRNA expression of airway markers (P63, MUC5AC and SCGB1A1). Groups were compared using one-way analysis of variance followed by Dunnett’s multiple comparisons test. *p<0.05, **p<0.01 and ****p<0.0001. The experiments were repeated at least three times. (B) Immunofluorescence images showing the expression of basal cell marker P63 (green), goblet cell marker MUC5AC (red), and club cell marker SCGB1A1 (green) in hLOs. (TIF) [file ppat.1012295.s001.tif]

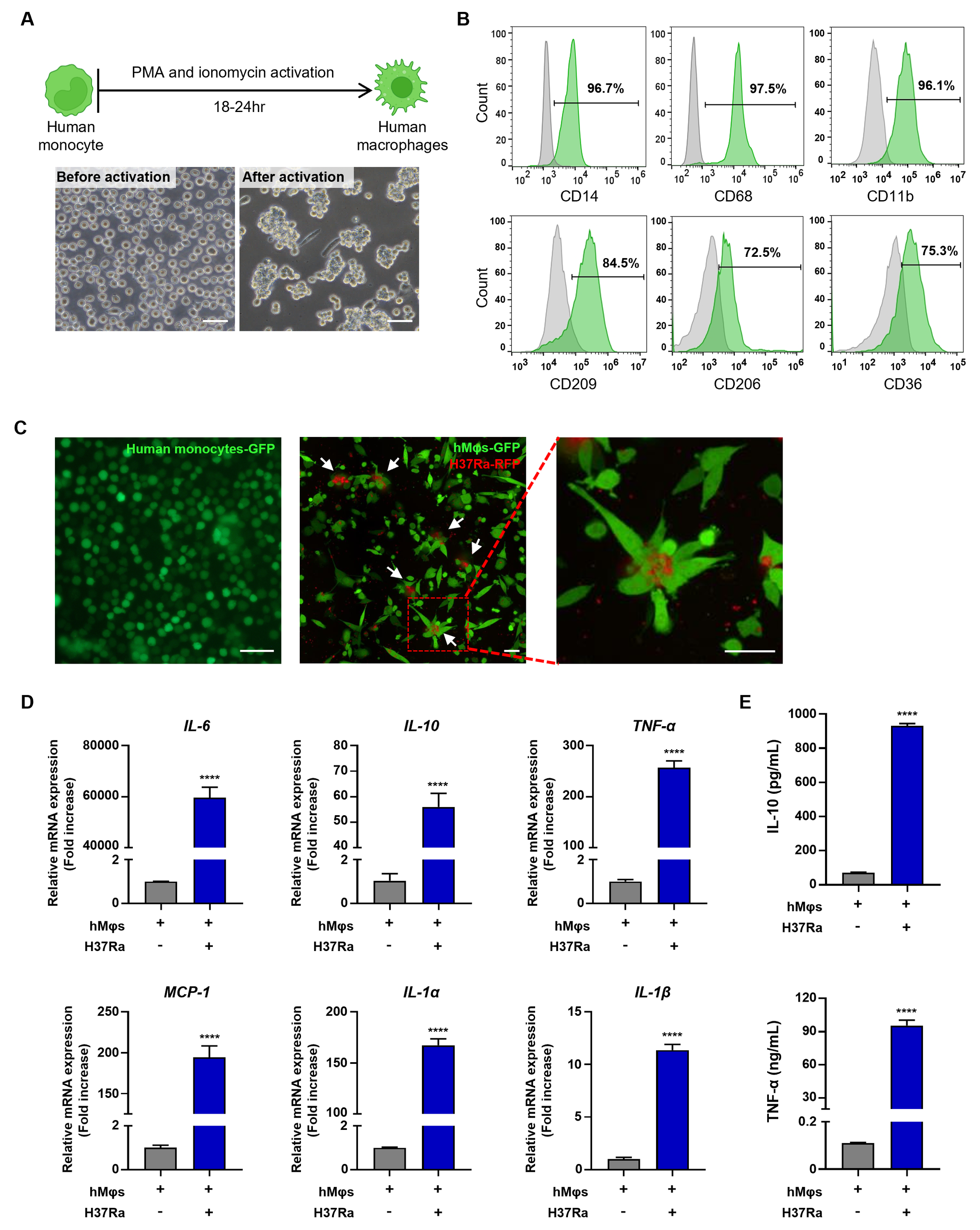

Supplement: S2 Fig — (A) GFP-expressing human monocytes were activated by PMA/Ionomycin cocktail to induce monocyte-derived Mφs for 24 h. Representative images show changes in the morphology of monocytes. Figure Created with BioRender.com. (B) Some macrophage-specific markers (CD14, CD68, CD11b, Cd209, CD206, and CD36) were determined by flow cytometry after activation. Grey histograms represent isotype control. (C) Fluorescence microscopy images of induced cell aggregation in M.tb H37Ra-infected Mφs. (D) Relative mRNA expression levels of cytokines (IL-6, IL-10, TNF-α, MCP-1, IL-1α, and IL-1β) and (E) quantification of cytokine protein expression (IL-10 and TNF-α) produced by M.tb H37Ra-infected Mφs (MOI = 5). The experiments were repeated at least three times. Statistically significant differences were determined using an unpaired two-tailed t-test. ****p < 0.0001. Scale bars, 50 μm. (TIF) [file ppat.1012295.s002.tif]

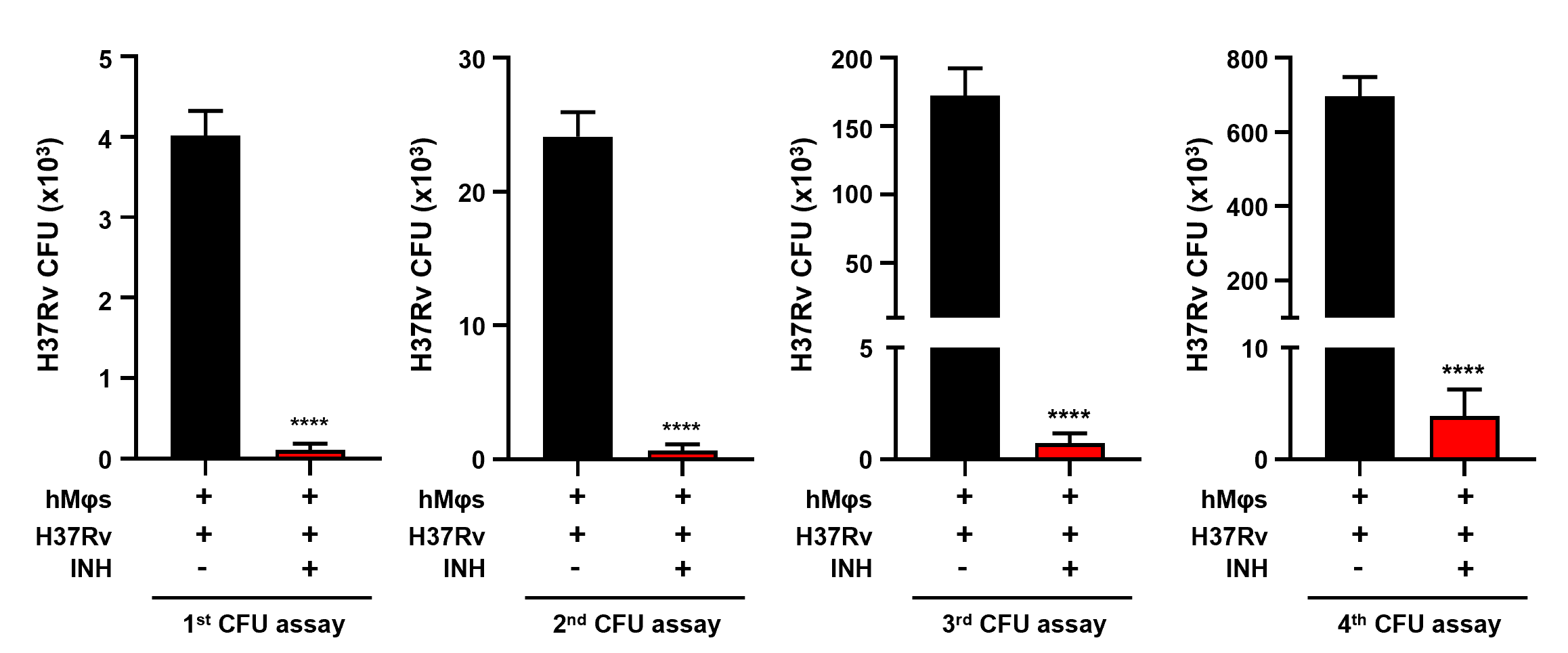

Supplement: S3 Fig — Intracellular survival of M.tb H37Rv in hLOs using CFU assay at each passage followed by post-treatment with anti-TB drug (INH). M.tb H37Rv infection model was treated with 10 μg/mL INH for 48 h before CFU assay. The experiments were repeated at least three times. Statistically significant differences were determined using unpaired two-tailed t-test. ****p < 0.0001. (TIF) [file ppat.1012295.s003.tif]

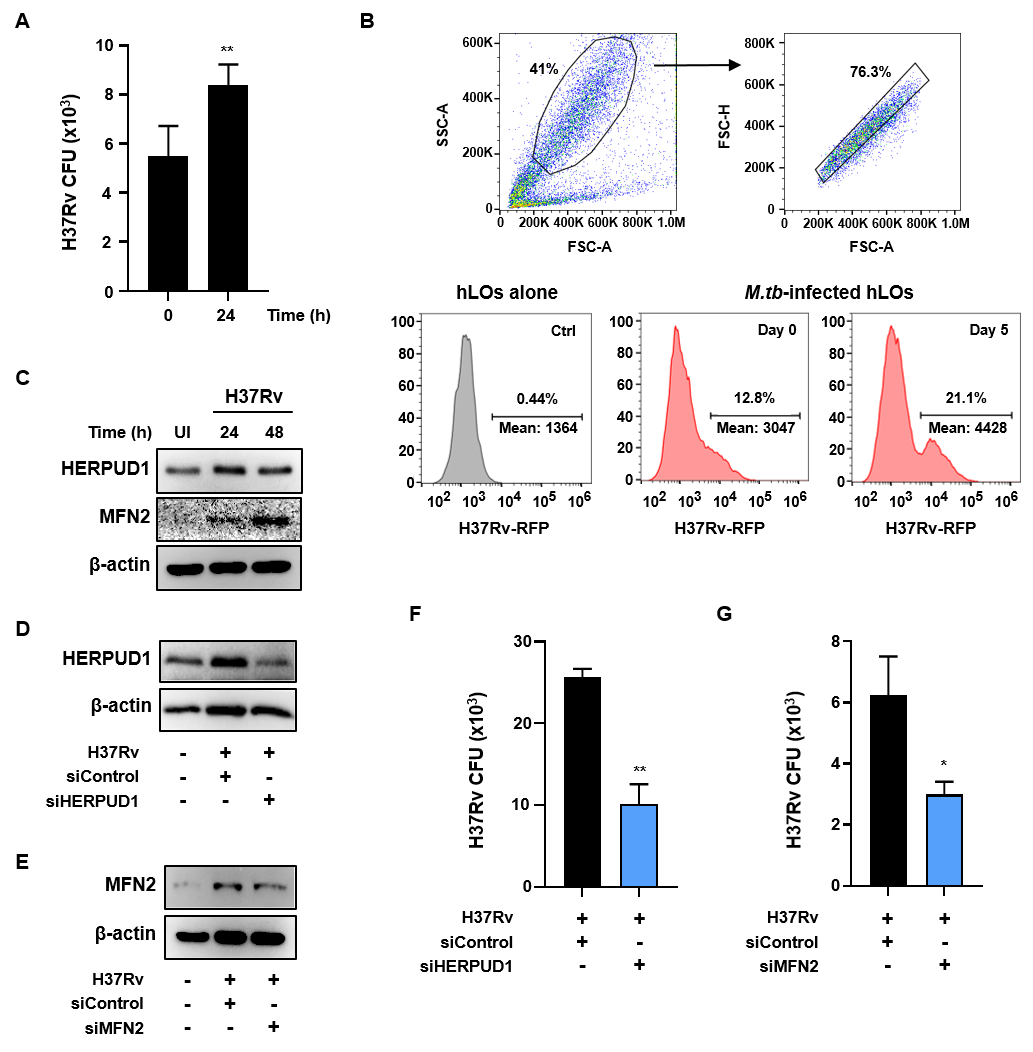

Supplement: S4 Fig — (A) Human epithelial cell line, BEAS-2B cells were infected with M.tb H37Rv (MOI = 1) for 24 h. Intracellular survival of M.tb H37Rv was assayed by CFU analysis. (B) Single-parameter flow cytometry histogram of RFP. hLOs were infected with M.tb H37Rv (MOI = 1) for 5 days. Upper panel shows gating strategy for flow cytometry analysis and lower panel shows the viability of M.tb H37Rv inside hLOs was detected by flow cytometry. Cells were selected from a FSC-A and SSC-A dot plot, and then gated in a FSC-A and FSC-H dot plot to eliminate doublets. Singlet cells were further analyzed for the expression of H37Rv-RFP to estimate M.tb growth. FSC-A, forward scatter area; SSC-A, side scatter area; FSC-H, forward scatter height. (C) Protein level of HERPUD1 and MFN2 was detected by western blot. UI, uninfected control. (D-G) BEAS-2B cells were transfected with specific siRNA for HERPUD1 or MFN2 (200 nM) and then infected with M.tb H37Rv (MOI = 1) for 48 h. Protein level of HERPUD1 or MFN2 and intracellular survival of M.tb H37Rv were analysis at 48 h after M.tb H37Rv infection. The experiments were repeated at least three times. Statistically significant differences were determined using unpaired two-tailed t-test. *p<0.05 and **p<0.01. (TIF) [file ppat.1012295.s004.tif]

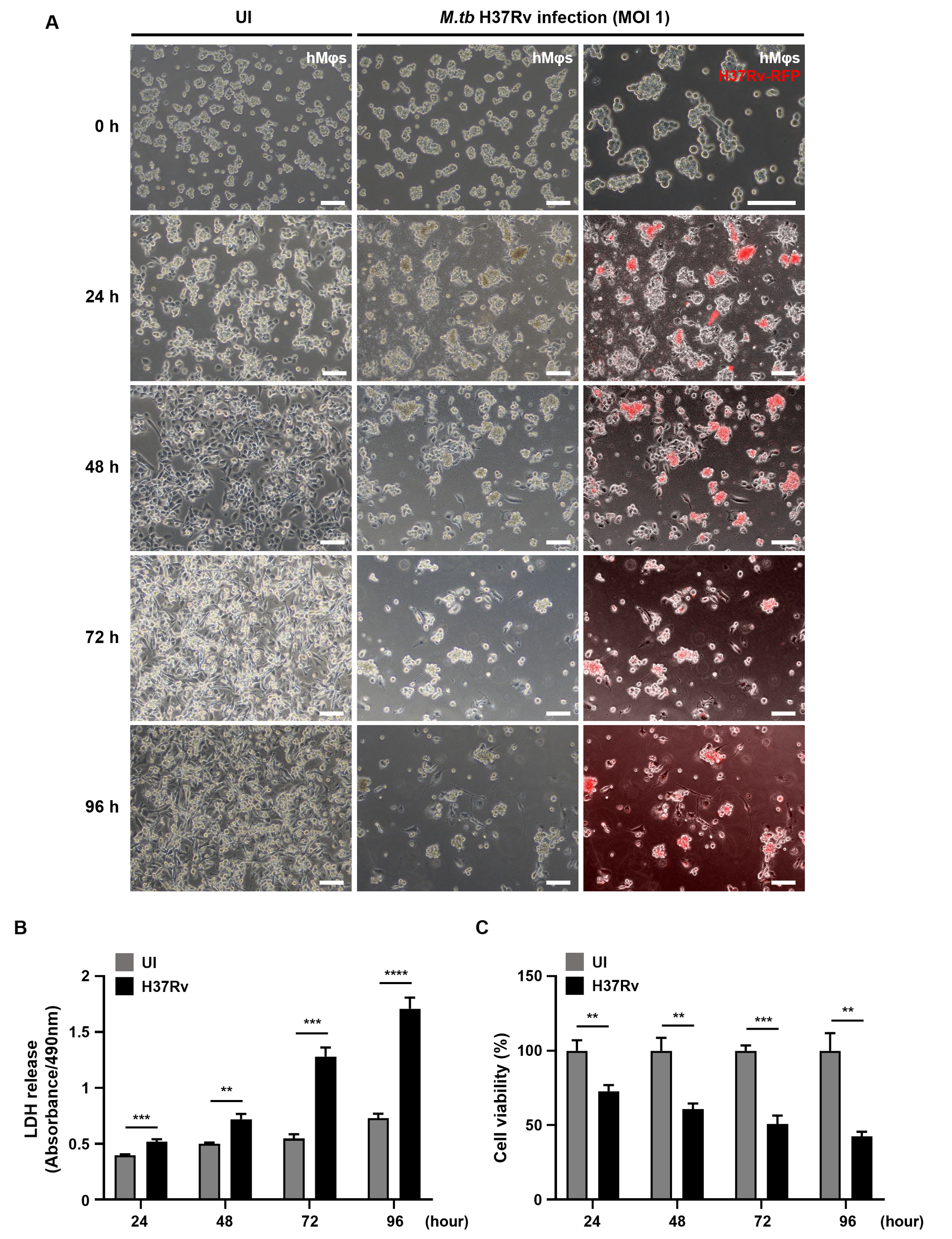

Supplement: S5 Fig — Human monocyte-derived macrophages (THP-1 cells) were infected with M.tb H37Rv (MOI = 1). Cell viability under M.tb infection was routinely monitored for 96 h through (A) fluorescence microscopy, (B) LDH assay, and (C) CCK-8 assay. The experiments were repeated at least three times. Statistically significant differences were determined using an unpaired two-tailed t-test. ****p<0.0001, ***p<0.001 and **p<0.01. Scale bar, 100 μm. UI, uninfected control. (TIF) [file ppat.1012295.s005.tif]

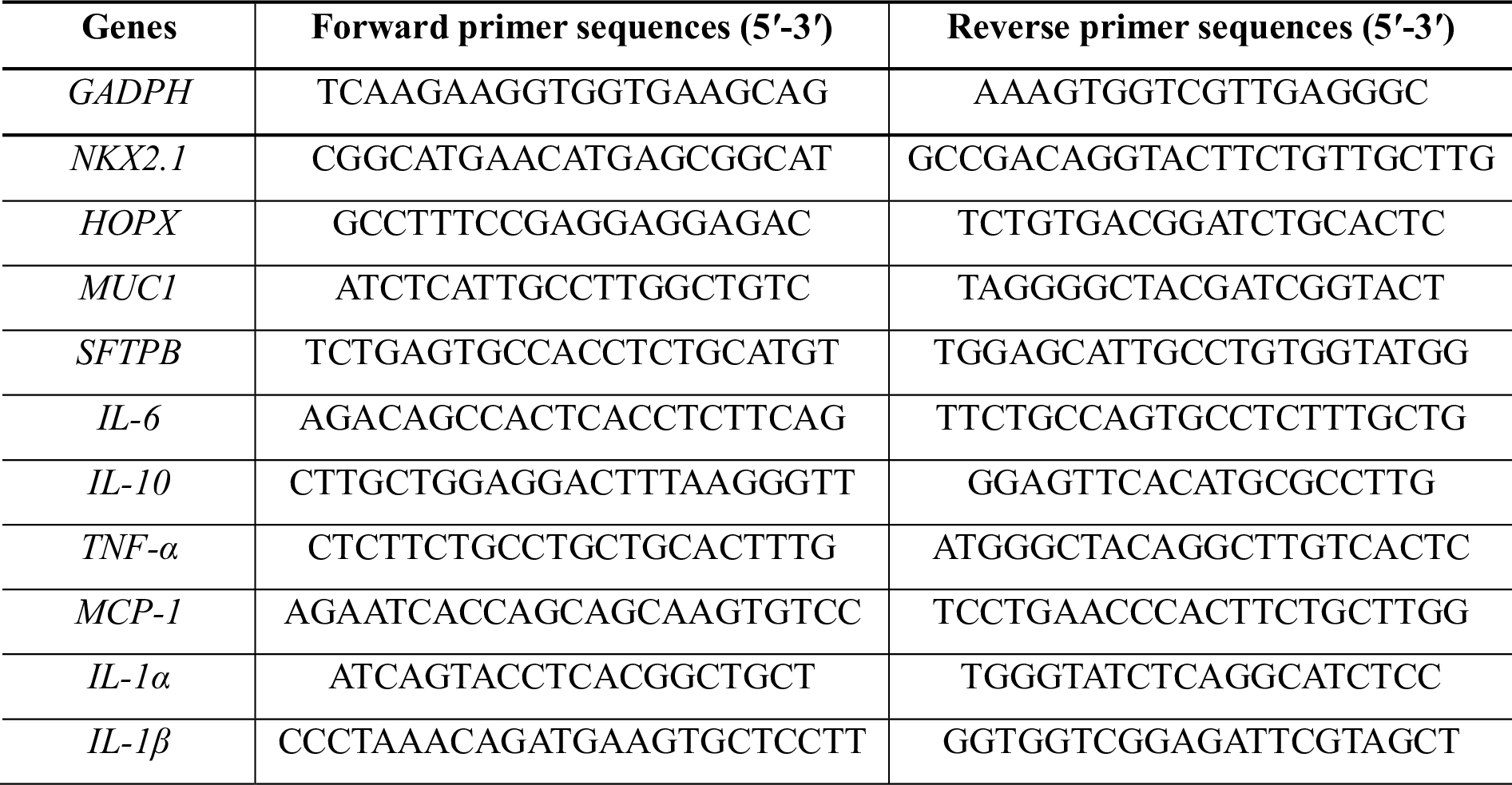

Supplement: S1 Table — (TIF) [file ppat.1012295.s006.tif]

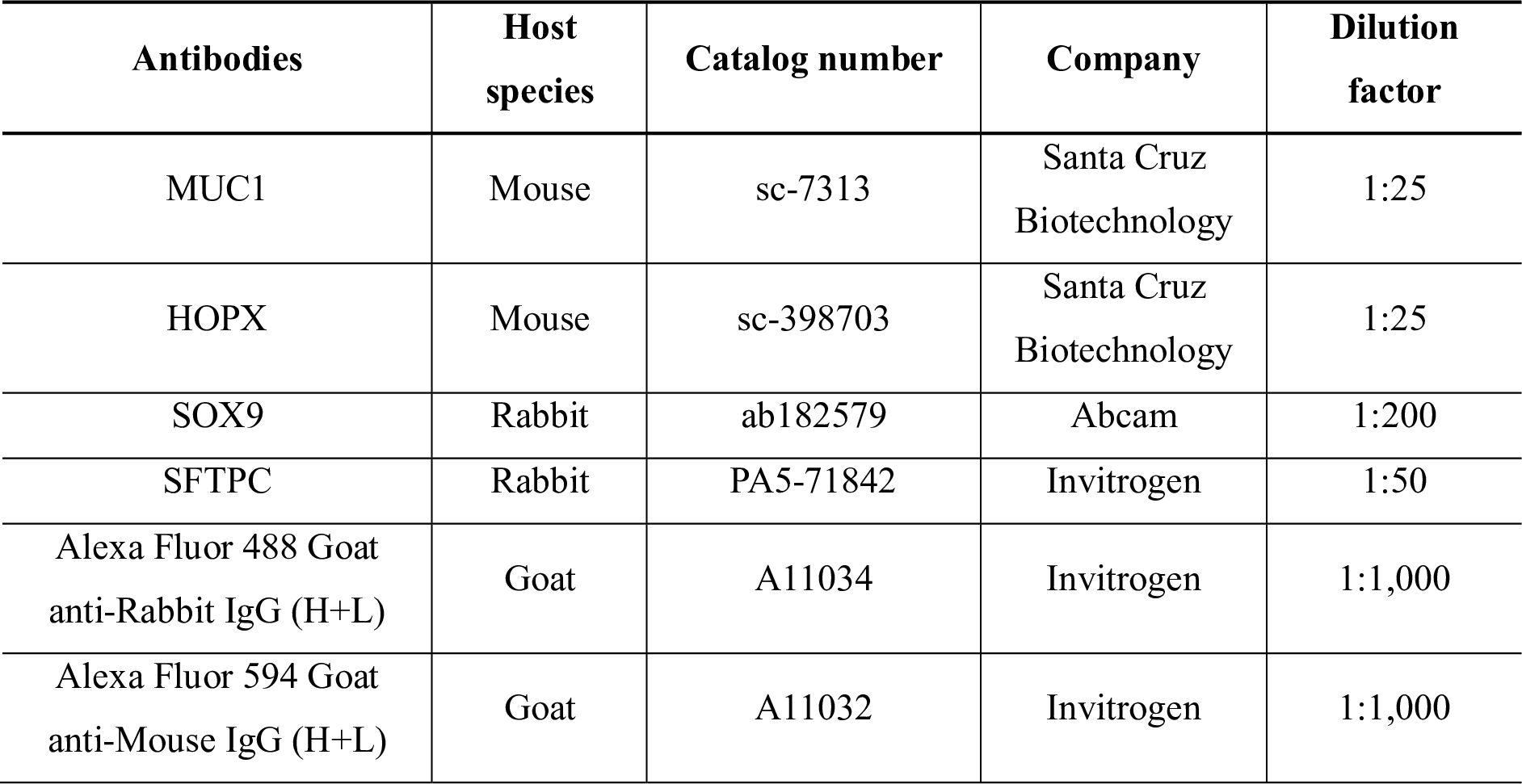

Supplement: S2 Table — (TIF) [file ppat.1012295.s007.tif]

Fig 6B.

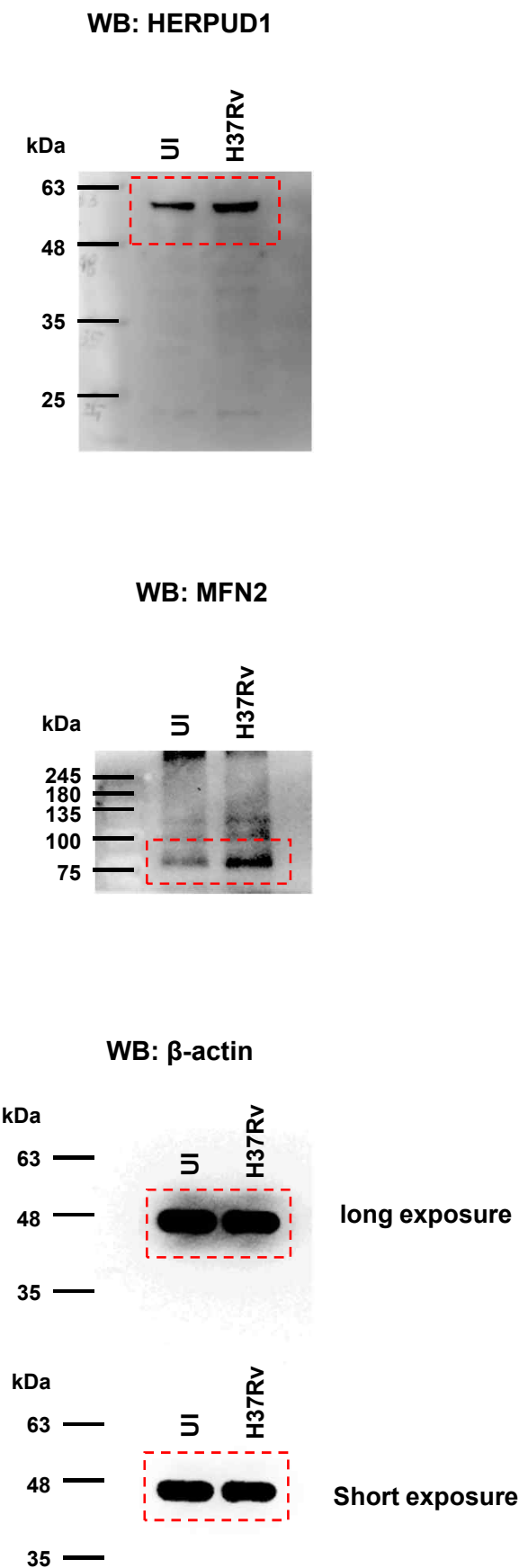

Fig 6C.

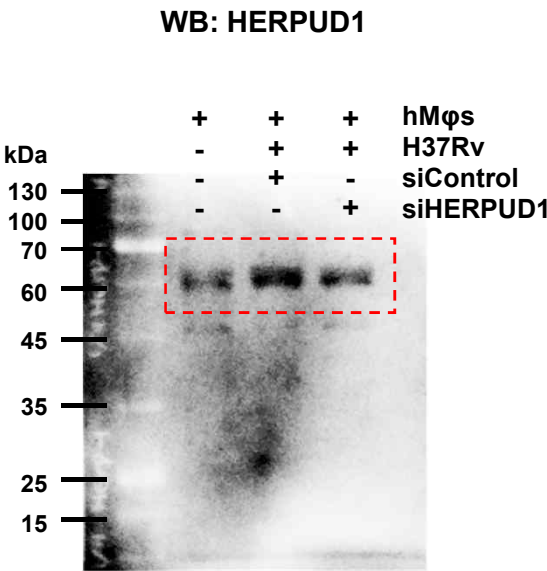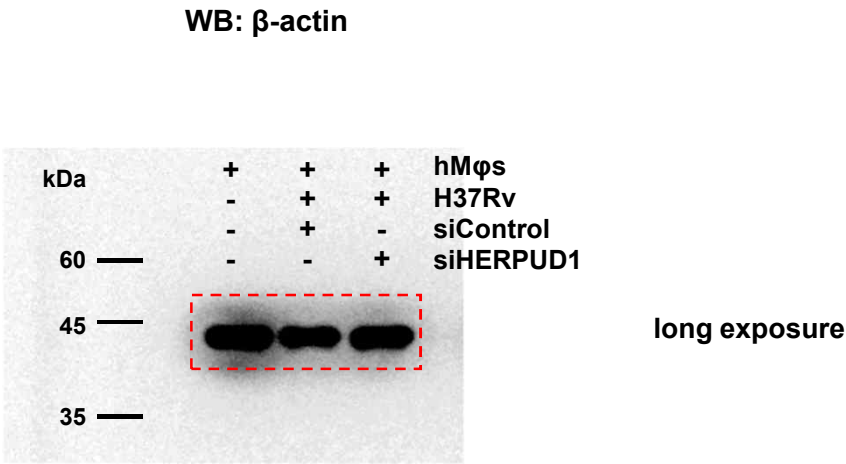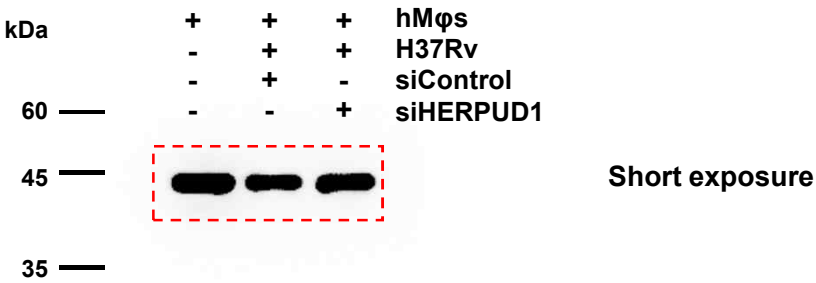

Fig 6D.

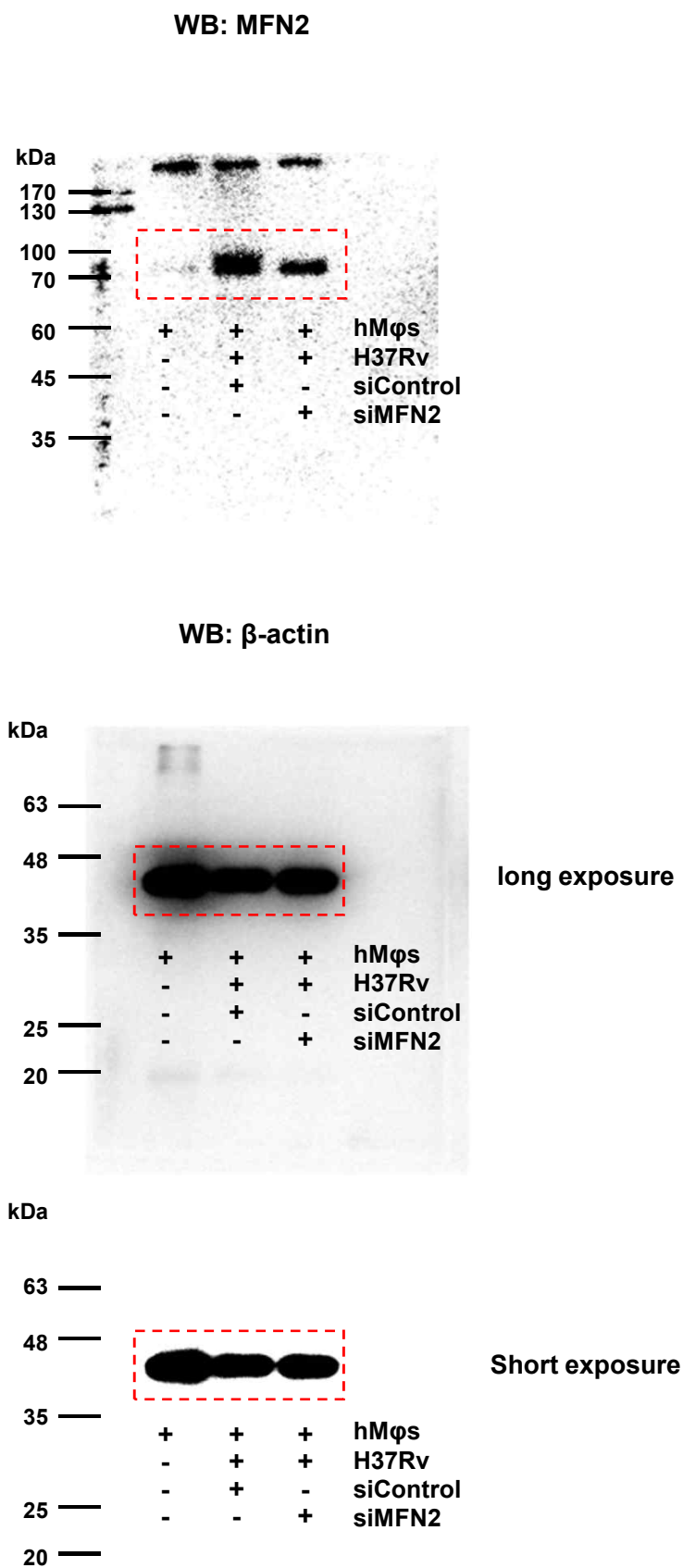

S4C Fig.

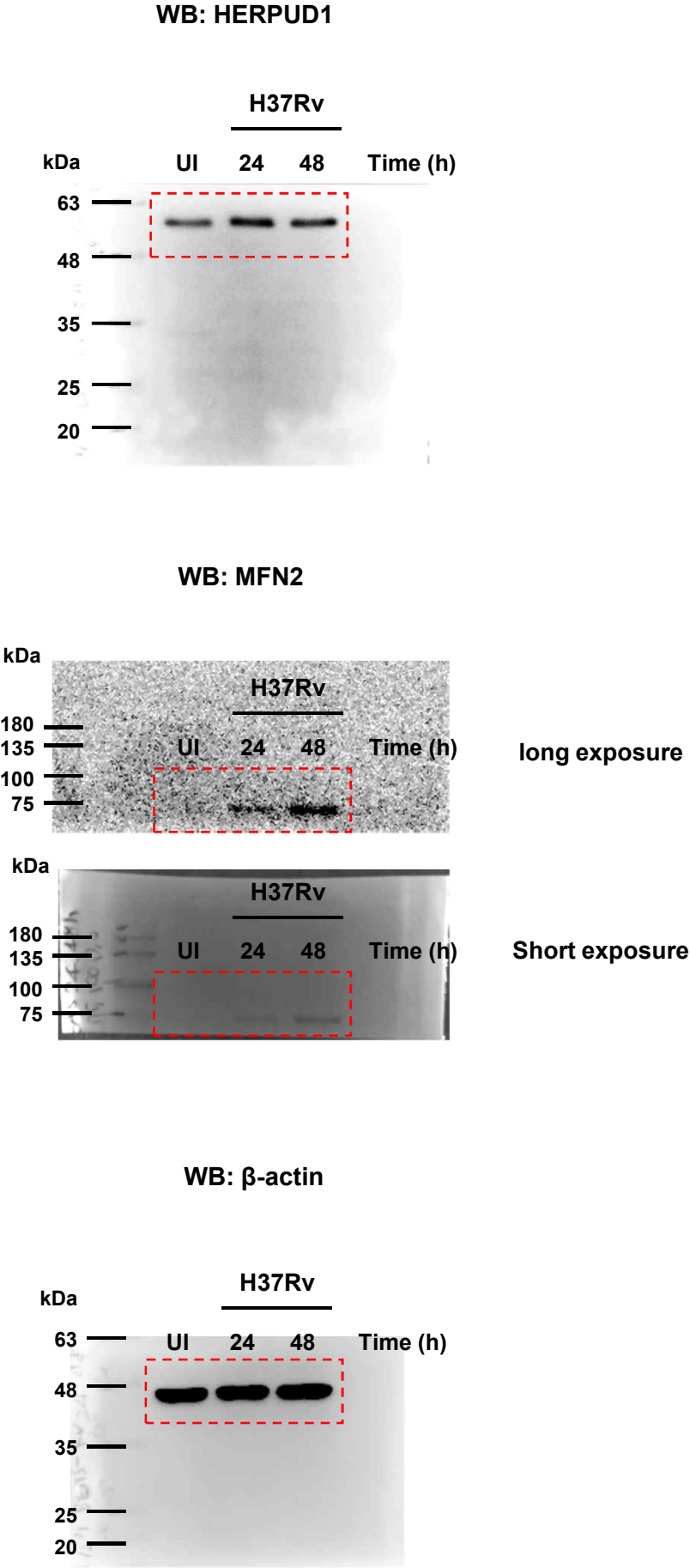

S4D Fig.

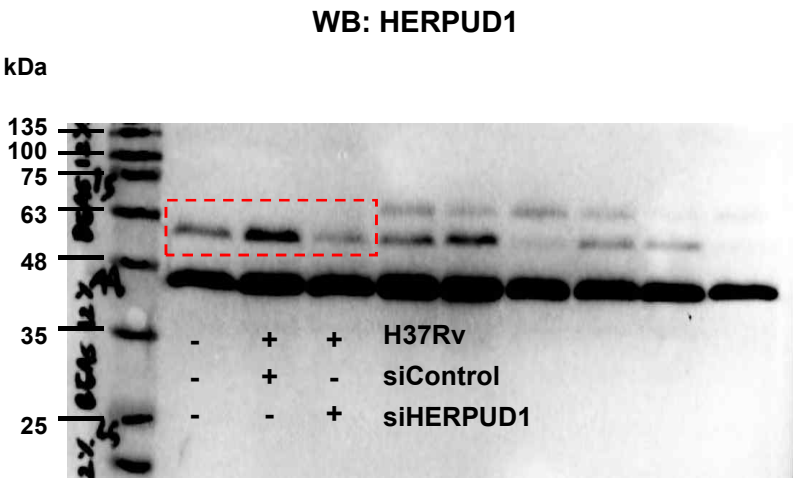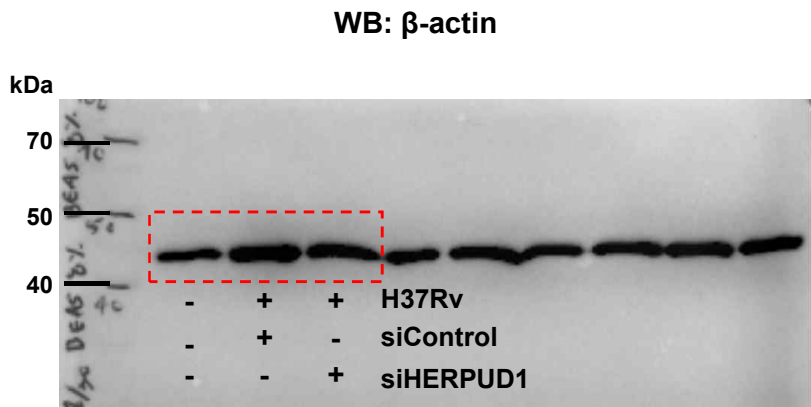

S4E Fig.

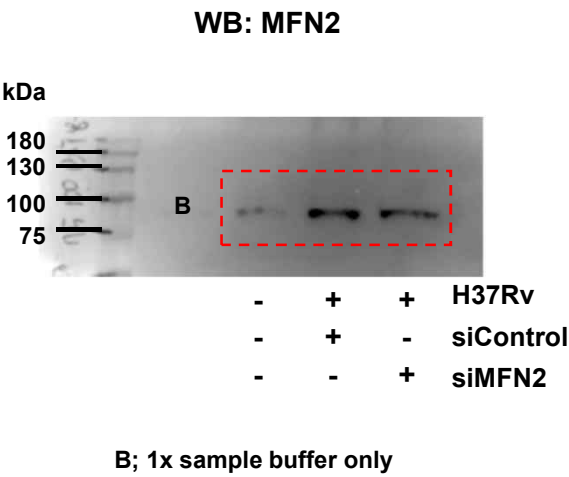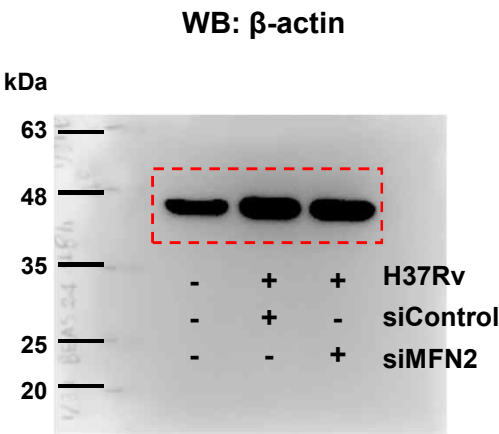

Supplement: S2 Data — (PDF) [file ppat.1012295.s010.pdf]
